# Interventions for reaching men to improve HIV Testing Services in sub-Saharan Africa: A narrative review

**DOI:** 10.4102/phcfm.v17i1.4869

**Published:** 2025-06-25

**Authors:** Lebogang G. Matonyane, Andrew Ross, Sandra Qolesa, Zandile Sibeko

**Affiliations:** 1 Department of Health Sciences, Faculty of Public Health and Family Medicine, University of KwaZulu-Natal, Durban, South Africa

**Keywords:** intervention, reaching, men, HTS, treatment uptake

## Abstract

**Background:**

The HIV Testing Services (HTS) are a vital component of human immunodeficiency virus (HIV) prevention initiatives, and the essential first step to healthcare. Men in South Africa have been shown to test for HIV at a lower rate than women, with a resulting higher mortality rate.

**Aim:**

This narrative review aimed to describe the approaches used to improve the uptake of HTS by men both at the facility and community level in sub-Saharan Africa (SSA).

**Method:**

Online databases were used to search for relevant studies published from 2019 to 2024 in English. A total of 475 records were identified, with 426 being included after duplicates were removed. After reviewing the abstracts, only 13 studies were included in the review.

**Results:**

This findings revealed three themes and seven sub-themes related to improving HTS uptake: improved access to testing (HIV self-testing, community-based testing and workplace testing), motivation and support (stakeholder involvement and creating a demand for testing) and health facility services (facility-based testing and services provided by male healthcare workers).

**Conclusion:**

The limited number of studies highlights the need for more research into developing and testing interventions to encourage men to utilise HTS. A multipronged approach that includes various role-players can be beneficial. This needs to be supported by continuous demand creation utilising platforms such as social media, radio and local newspapers.

**Contribution:**

The study collates the interventions intended to encourage men to undertake HTS in SSA.

## Introduction

Acquired immunodeficiency syndrome (AIDS) is a disease of global public health importance, and contributes to high morbidity and premature mortality rates.^1^ Susceptibility to human immunodeficiency virus (HIV) infection is often because of vulnerable behaviour, which can affect all people, regardless of class, gender, race or ethnicity, sexual orientation and age.^2^ In 2023, an estimated 18.1 million men aged 15 years and older were living with HIV in the world including men who have sex with men (MS M).^3^ Based on the mentioned statistics, it is estimated that globally 39.0 million people were living with HIV (PLHIV) in 2022, of whom 48% were men, with 29.8 million accessing antiretroviral therapy (ART).^3^ Across sub-Saharan Africa (SSA), in 2021, only 73% of males with HIV aged 15 years or older were aware of their status, compared to 83% of women in the same age range.^4^

The HIV Testing Services (HTS) are a vital component of HIV prevention initiatives and the essential first step in getting individuals into treatment, with men in South Africa having been shown to test for HIV at a consistently lower rate than women.^5,6,7,8^ While women bear the highest HIV/AIDS burden of disease in SSA,^1^ mortality rates for men are higher than for women, with contributory factors including poor health-seeking behaviour, low treatment coverage, poor adherence, late presentation and high rates of lost-to-follow-up (LTFU).^1,9^

According to the South African National Department of Health (NDoH) (2023), HTS should be made available in all public health facilities, community settings, private healthcare facilities and non-governmental organisations (NGOs) that have been approved to offer HTS, this is sometimes not the case.^10^ In all settings, it is the duty and responsibility of all healthcare and health auxiliary workers to inform people about the risks of HIV to encourage them to make informed decisions about testing.^11^ Healthcare workers (HCWs) are encouraged to offer HIV testing to all patients to identify those who are positive including men, their partners, HIV-exposed and HIV-positive infants, children and youth, to ensure timeous treatment.^11^

Poverty can also directly affect willingness to test and engagement in care, as individuals lack the resources required to attend clinics (cost of transport), additional barriers to visiting facilities including confidentiality concerns, long waiting time, loss of wages, inconvenient hours and the perception that clinics are places for women.^12,13,14^ A realistic and practical approach to health promotion that encourages and empowers men to put their health first is therefore required to improve HTS among males in the SSA region.^6^ To increase the number of men who receive HIV testing, there needs to be a focus on the use of resources and channels that are not part of the traditional healthcare system. This implies that men’s uptake might increase if HTS were offered in casual settings, such as informal places where they gather, on the farms where they live and work and at soccer tournaments.^7^ To address the challenges relating to access to comprehensive HTS, including HIV prevention and ART, targeted interventions need to be strengthened and continuously monitored for impact.

This narrative literature review therefore aims to identify key interventions that have been implemented across and beyond healthcare systems to improve HTS uptake among men that may improve their access to HIV prevention services, such as Pre-Exposure Prophylaxis (PrEp), condoms and health promotion. This will be done by identifying those interventions that can be scaled up among men to enhance their HTS uptake, these being essential for improving their health and well-being, enabling them to learn about their HIV status timeously and make informed decisions regarding their health.

## Methodology

The most influential publications that elucidate and debate the current state of science on a particular subject or theme, from a theoretical and contextual perspective, are regarded as appropriate narrative literature review articles as stated by Juntunen and Lehenkari.^15^ The most recent literature on the subject was identified to serve as the foundation for this review, that being within the last 5 years.^15^ To address the aims of this article, the three phases of reporting, conducting and planning, and nine steps of a narrative literature review were followed as indicated in Table 1.

**TABLE 1:** Narrative literature review steps.

Phase 1. Planning	Phase 2. Conducting	Phase 3. Reporting
1. Select the topic	6. Analyse	8. Conclude
2. Define	7. Synthesise	9. Report
3. Develop	-	-
4. Search	-	-
5. Select the literature	-	-

*Source*: Adapted from Juntunen M, Lehenkari M. A narrative literature review process for an academic business research thesis. Stud High Educ. 2021;46(2):330–342. https://doi.org/10.1080/03075079.2019.1630813

### Phase 1. Planning

This phase covers topic selection, identifying the research objective and question, and setting out the procedure to be followed to search for the literature through the first 5 steps.

#### Step 1: Select the topic

The topic for this study is interventions for reaching men through HIV testing opportunities to improve access to treatment services.

#### Step 2: Define the aim and objectives and formulate the research questions

The study’s aim is to describe the interventions that will improve the uptake of HTS opportunities for men at both facility and community levels to improve their access to treatment services. The research question to be addressed was, what interventions have been implemented for improving the uptake of HTS which would then facilitate improved access to treatment services for men?

#### Step 3: Develop and validate a review protocol

This step entailed developing a plan regarding how to undertake the subsequent steps of the research process, being equivalent to research design in empirical research. The first author conceptualised the study, while the protocol was validated by other authors involved in the project. L.G., S.Q. and Z.S. were responsible for the entire study process, through conceptualisation, methodology design, the conduct of the research and project management, data analysis and validation. A.R. was the overall study supervisor and contributed to the article’s idea, method design, validation and critical review.

#### Step 4: Search the literature

Two reviewers searched for studies from three online databases, these being Google Scholar, Science Direct and the World Health Organization (WHO) website. The search terms used to identify relevant articles included intervention, reaching, men, HTS and treatment access. The inclusion criteria were recent studies conducted from 2019 to 2024 that were published in English, with comprehensiveness and relevancy of the literature being prioritised, with those published before 2019 being excluded. Those involving the general population or with a focus on women were excluded, as were newspapers, conference articles and other databases.

#### Step 5: Select the literature: You need to select the search words before you can search

Of the 475 records identified through database searching, 426 articles were included after duplicates were removed, of which 240 were excluded after reviewing the abstracts, and 173 full-text articles were excluded because of other exclusion criteria. This resulted in 13 studies being included in this narrative literature review.

### Phase 2. Conducting

This phase covers the analysis of identified records and arranging the gathered data for the narrative review through two steps.

#### Step 6: Analyse

All authors graded the evidence from each report using the Johns Hopkins Nursing Evidence-Based Practice Research Evidence Appraisal instrument.^15^ The tool’s quality guidelines are represented by the letters A, B and C for High Quality, Good Quality and Low Quality or Major Flaws, respectively. The year, purpose, design, sample and outcomes determined the study’s quality.^15^

#### Step 7: Synthesise

The gathered data were arranged according to a predetermined structure.^15^ The independently collected data, underwent a preliminary thematic analysis by each author, after which they met to discuss and finalise the themes.^15^ The study’s findings identified 3 themes, 7 sub-themes and 17 categories from the literature.

### Phase 3. Reporting

This phase addresses recommendations for future research and consisted of two steps.

#### Step 8: Conclude

The findings provide opportunities to strengthen existing strategies and offer recommendations for further study to broaden the body of knowledge on the subject relating to improving access to HTS among men in SSA. The authors made recommendations that healthcare providers could implement and acknowledged the study’s limitations.

#### Step 9: Report

This literature review is organised as per Juntunen and Lehenkari’s structure: title, abstract, introduction, design and methods, discussion, conclusion and list of references.

### Ethical considerations

In this review, no Biomedical Research Ethics permission was required as this was a review of already published studies. The methodology section outlines the contributions made by each author in writing this article, their varying backgrounds enhancing the study’s credibility.

## Review findings

**FIGURE 1:**
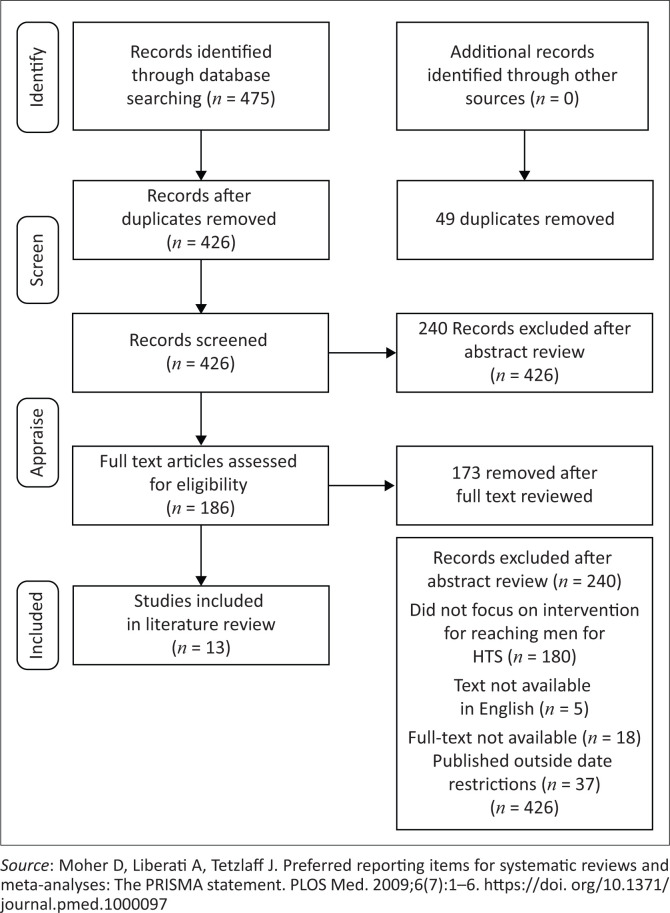
Flow chart of the search strategy.

Figure 1 displays the search strategy used to identify, screen and apprise the literature that was sourced for the study; this process resulting in 13 articles being included.

Table 2 shows the included studies, their aim and objective, study design, population and context, data collection and analysis methods, and the main findings. Of the 13 studies included, 7 were qualitative, 5 were quantitative and 1 was policy brief from the WHO. While 2 studies included adolescent boys and young men (ABYM), 10 focused on the general male population, with 9 being conducted in Southern Africa and three in Eastern Africa.

**TABLE 2:** Studies accessed for narrative literature review.

No	Study	Variables	Study details
1.	Adeagbo O, Xulu Z, Gumede D, Naidoo K. 2024. Barriers and strategies to improve men’s uptake of HIV care services in rural KwaZulu-Natal, South Africa: A qualitative approach.	Design	Descriptive exploratory qualitative study design
		Population and context	Black African males, rural KwaZulu-Natal province, South Africa,
		Data collection	10 telephone in-depth interviews (IDIs) and 3 participatory workshops (PWs)
		Data analysis	Thematic analysis
		Findings	Participants noted that men need personalised HIV/AIDS messaging and education, led by men living with HIV, about the benefits of HIV testing, treatment and prevention
2.	Cassidy T, Cornell M, Makeleni B, Horsburgh CR, Duran LT, Azevedo VD, Boulle A, Fox MP. 2022. Attrition from care mong men initiating ART in male-only clinics compared with men in general primary health care clinics in Khayelitsha, South Africa: A matched propensity score analysis.	Design	Propensity score matched cohort study
		Population and context	Primary Health care Clinics in Khayelitsha, Cape Town, South Africa
		Data collection	Routine patient data collected from folders
		Data analysis	Chained multiple imputation was performed using the ice procedure in Stata
		Findings	Male-only clinics reached younger, healthier men, and had lower ART attrition than general services. These findings support clinic-specific adaptations to create more male-friendly environments
3.	Conserve DF, Issango J, Kilale AM, Njau B, Nhigula P, Memeah P, Mbita G, Choko AT, Hamilton A, King. 2019. Developing national strategies for reaching men with HIV testing services in Tanzania: Results from the male catch-up plan.	Design	Descriptive qualitative study design
		Population and context	Tanzania, Dar es Salaam, men, health care workers, secondary school boys and members of the community
		Data collection	In-depth interviews
		Data analysis	ATLAS.ti 7.5 qualitative analysis software
		Findings	A number of barriers, fear of testing positive, high HIV risk perception and low HIV risk perception contribute to the low uptake of HTS among men in Tanzania. National strategies have been developed to address these HTS barriers and guide the national Test and Treat campaign focusing on increasing HTS uptake among men
4.	Deane D, Wamoyi J, Mgunga S, Changalucha J. 2022. HIV testing attitudes and practices amongst ‘wealthy men’: Qualitative evidence from Tanzania	Design	Descriptive qualitative study design
		Population and context	Mwanza City, Tanzania, 23 wealthy men
		Data collection	Semi-structured interviews
		Data analysis	Descriptive analyses
		Findings	In settings such as the workplace, wealthy men were able to conduct HTS in public in their roles as ‘leaders’ to encourage others to do the same.
5.	Gottert A, Pulerwitz J, Heck CJ, Shabangu D, Lukhele B, Cawood C, Khanyisile D, Apicella L, Okal J, Mathur S. 2022. Inroads for HIV prevention among men: Findings from mixed methods research in the context of the DREAMS partnership in Southern Africa	Design	Independent cross-sectional surveys
		Population and context	Eswatini and Durban, 74 men exposed to HIV services/prevention programming.
		Data collection	In-depth interviews
		Data analysis	Stata v16
		Findings	Qualitative data suggest HTS uptake was facilitated by increased convenience and supportive information/messaging about HIV treatment efficacy
6.	Jobson G, Khoza S, Mbeng R, Befula N, Struthers HE, Kerongo G, Peters RPH.2019. Bridging the Gap: Reaching Men for HIV Testing Through Religious Congregations in South Africa	Design	Descriptive quantitative study design
		Population and context	Rural Mopani District of Limpopo province, South Africa, 1971 church members
		Data collection	Assessment of the HTS intervention using routine programme data
		Data analysis	Descriptive analysis
		Findings	Religious institutions and faith-based organisations (FBOs) remain an important aspect of the response to the HIV epidemic in Africa and South Africa, and could potentially play a significant role in reaching populations with low levels of uptake of HTS
7.	Muwanguzi PA, Nasuuna EM, Namimbi F, Osingada CP, Ngabirano TM. 2021. Venues and methods to improve professional men’s access to HIV self-testing and linkage to HIV prevention or treatment: A qualitative study	Design	Explorative-descriptive qualitative study
		Population and context	33 men from 6 Ugandan urban centres.
		Data collection	In-depth interviews
		Data analysis	Thematic content analysis
		Findings	The process of HIV self-testing may be optimised by providing collection bins in designated areas, and using mHealth or mobile phone applications
8.	Ndlovu S, Ross A, Mulondo M. 2023. Interventions to improve young men’s utilisation of HIV-testing services in KwaZulu-Natal, South Africa: Perspectives of young men and health care providers	Design	A descriptive exploratory qualitative study
		Population and context	17 young men and 2 health care providers in Ladysmith, KwaZulu-Natal province
		Data collection	Semi-structured interviews
		Data analysis	Braun and Clarke’s six stages of thematic analysis
		Findings	Prioritising men in the morning at the primary health care facilities, and establishing male clinics within communities as key factors to improving the uptake of HTS among young men. An improvement in the health care provider attitudes and service delivery, establishment of adherence clubs for young people living with HIV, ensuring a diverse and balanced health care provider staff composition at primary health care facilities
9.	Nyirenda HC, Foloko M, Bolton-Moore C, Vera J, Sharma A. 2023. Drivers of uptake of HIV testing services, a snapshot of barriers and facilitators among adolescent boys and young men in Lusaka: A qualitative study	Design	Exploratory qualitative study design
		Population and context	24 adolescent boys and young men in Lusaka
		Data collection	Focus group discussion and In-depth interviews
		Data analysis	NVivo software V.12 (QSR International).
		Findings	Both groups agreed that Adolescent Boys and Young Men (ABYM) need services specifically designed for them that offered convenient, private, swift and non-judgemental services
10.	Nyondo-Mipando AL, Kumwenda M, Suwedi-Kapesa LC, Salimu S, Kazuma T, Mwapasa V. 2021. ‘You Cannot Catch Fish Near the Shore nor Can You Sell Fish Where There Are No Customers’: Rethinking Approaches for Reaching Men With HIV Testing Services in Blantyre Malawi	Design	Descriptive qualitative study
		Population and context	A total of 113 men of varying HIV statuses in 7 public health facilities in Blantyre, Malawi
		Data collection	A total of 20 in-depth interviews and 14 focus group discussions
		Data analysis	NVivo and followed a thematic approach in analysing the data
		Findings	HIV testing and counselling (HTC) uptake among men may be increased if the services were provided at informal places
11.	Phiri MM, Schaap AB, Simwinga M, Hensen B, Floyd S, Mulubwa C, Simuyaba M, Chiti B, Bond V, Shanaube K, Fidler S, Hayes R, Ayles H. 2022. Closing the gap: Did delivery approaches complementary to home-based testing reach men with HIV testing services during and after the HPTN 071 (PopART) trial in Zambia?	Design	Three-arm community randomised trial
		Population and context	Between 2013 and 2017, three intervention rounds (IRs) of door-to-door HTS delivery were conducted in eight PopART communities in Zambia
		Data collection	Intervention was delivered by a pair of trained CHWs covering a geographical area of ~450 households.
		Data analysis	Population enumeration data for each round of HTS provided the denominator, allowing for calculation of proportion of men tested as a result of each strategy during different time periods
		Findings	Achieving high coverage of HTS among men requires universal, home-based service delivery combined with an option of HIVST and delivery of HTS through community-based hubs
12.	Sithole N, Shahmanesh M, Koole O, Krows M, Schaafsma T, Siedner J, Celum C, Barnabas R, Shapiro AE. 2021. Implementation of HIV self-testing to reach men in rural uMkhanyakude, KwaZulu-Natal, South Africa. A DO-ART trial sub study	Design	Three-arm community randomised trial
		Population and context	A total of 99 men in the uMkhanyakude district, KwaZulu-Natal
		Data collection	During the distribution period (August–November 2018), three teams of a nurse and clinical research assistant and four recruiters canvassed the district to introduce and distribute HIVST kits to men as an alternative way to test for HIV
		Data analysis	Multivariate regression models in R (Version 4.0) to identify predictors for those who reported back their HIVST results
		Findings	Large scale distribution of HIVST kits targeting men in rural KwaZulu-Natal is feasible and highly effective in reaching men, including those who had not previously tested for HIV
13.	WHO. 2021. Improving men’s uptake of HIV testing and linkage to services	Design	Policy brief
		Population and context	HIV Testing Services
		Data collection	There were no data collection for the policy brief
		Data analysis	There were no data analysis as this was a policy brief.
		Findings	HTS can link men to these services as appropriate for their test results

*Source:* Adapted from Sharma M, Barnabas RV, Celum C. Community-based strategies to strengthen men’s engagement in the HIV care cascade in sub-Saharan Africa. PLoS Med. 2017;14(4):e1002262. https://doi.org/10.1371/journal.pmed.1002262

## Themes

The thematic analysis of the included articles revealed three main themes: (1) Improved access to testing; (2) Motivation and support; and (3) Health facility services. The three main themes, the seven sub-themes and their associated categories are indicated in Table 3.

**TABLE 3:** Themes and sub-themes.

Themes	Sub-themes	Categories
1. Improved access to testing	1. HIVST	Home-based HIVST Community-based HIVST Workplace HIVST Facility-based HIVST
	2. Community-based testing	Hotspots testing Door-to-door testing (Home-based testing) Mobile clinics
	3. Workplace testing	Industrial or workplace-based testing Maintain confidentiality and avoid stigma
2. Motivation and support	1. Stakeholders involvement	Faith-based organisations Men’s health associations
	2. Create a demand for testing	Motivate and encourage men to conduct testing Share information about the importance of HTS
3. Health facility services	1. Facility-based testing	Offer provider-initiated testing and counselling Provide comprehensive HTS during facility visit
	2. Services provided by male healthcare workers	Provide male friendly health services Provide services by male nurses and counsellors

HTS, HIV testing services; HIV, human immunodeficiency virus; AIDS, acquired immunodeficiency syndrome; HIVST, HIV self-testing.

### Theme 1: Improved access to testing

This section consisted of three sub-themes: HIV self-testing, community-based testing and workplace testing. Routine HIV testing should be offered to all clients in all clinical settings to improve uptake, with active linkage to prevention, treatment and care services, in addition to routine facility-based and workplace testing.^4^

#### Sub-theme 1: HIV self-testing

Five studies (3,7, 11, 12 and 13, refer to Table 2) referred to HIV self-testing as a key strategy in improving HTS coverage among men, and was found to have been conducted in various places. This being addressed through four categories of home-based, community-based, workplace model and facility-based HIV self-testing (HIVST).

The HIV self-testing entails a person obtaining a test from their local clinic, being shown about how to use it, counselled about the implications of testing positive and informed about the need for regular testing if they are negative.^4^ The test entails collecting their specimen (oral fluid or blood), performing the test using a kit onto which the specimen is dropped and interpreting the result based on the line that appears to denote the presence or absence of the virus.^4^ The HIV self-testing may be convenient for users as it displays the result within a few minutes, having the potential to reduce the number of facility visits and eliminate the need for individuals to travel long distances or wait in long lines to access HIV testing. This means that people can conduct a test at a chosen space after the demonstration by the HCWs, and they only need to return to the clinic if the test is positive in order to access ART.^16^

The latest evidence indicates that HIVST increases the rate of HIV testing among men, including those from key populations, such as sex workers and people who inject drugs, and is comparable to facility-based testing regarding reliability.^4,17^ A study conducted in South Africa with the large-scale distribution of HIVST kits to men in rural KwaZulu-Natal province demonstrated it to be both feasible and highly effective in reaching this cohort, including those who had not tested before.^16^ While two-thirds of individuals who tested positive for HIV had started ART, the authors contend that it is important to develop additional strategies to ensure that those who test positive but do not go to the clinic for further testing to confirm the results and access ART are effectively supported.^16^

**Home-based HIV self-testing:** A study conducted between 2013 and 2017 in eight HIV Prevention Trials Network (HPTN) 071 (PopART) communities in Zambia found that achieving high testing coverage among men requires access to home-based service delivery through HCWs, combined with the option of HIVST and HTS.^17^ The study also revealed that men are willing to test for HIV when reached, but reaching them necessitates adapting the delivery strategies to enable them to access HIVST in a manner that meets their needs.^17^ A quantitative study showed that men who had discussed HIV testing with their partners were more likely to be willing to self-test, particularly those who had never tested before.^18^

This suggests that there is the potential for sexual partners to provide HIVST kits to men, including those who have never been tested, while engaging in conversations about HTS.^4,18^ Intensive door-to-door (home-based) HTS and revisits to households where the inhabitants use HIVST have the potential to reach more men than campaigns or workplace strategies.^17^

**Community-based HIV self-testing:** The HIV self-testing delivery models need to be adapted, with the engagement of communities, to reach men in their local context, with community-led models possibly being more acceptable.^4^ In a qualitative study involving 33 men from 6 Ugandan urban centres, researchers recommend using various methods to encourage their uptake of HIVST, which entailed distributing test kits to leisure, hotspots and online groups. The study found that involving female partners, peers and established men’s groups can help improve HIVST uptake.^19^ The research conducted for the Tanzania Self-Testing Education and Promotion (STEP) project revealed that men are open to self-testing for HIV, as they found it to be convenient and felt that it could assist in overcoming the barriers associated with accessing facility-based testing.^18^

**Workplace HIV self-testing:** Distribution of HIVST kits can be useful in high HIV burden settings where most of the employees are men.^4^ Partnerships with medical insurers may also be important for engaging men in professional employment in HIV services, including the use of HIVST.^19^ Trained workplace peers can be useful to promote, demonstrate and distribute HIVST kits to reach more men.^4^ Digital technology and mobile phones have been effectively used to facilitate the return of test results and to strengthen linkage to care or prevention services.^19^ An enabling environment to support stigma-free HIVST, including workplace policies that protect workers’ rights and confidentiality, is essential.^4^

**Facility-based HIV self-testing:** Secondary distribution of HIVST kits should include partners or peers, for example, women attending antenatal care (ANC) and index clients to their partners, and this could be useful for reaching men.^4^

The HIV self-testing is another strategy that has shown success in reaching high-risk men and first-time testers, hence their intensive distribution in healthcare facilities needing to be prioritised.^19^

#### Sub-theme 2: Community-based testing

Four studies (1, 3, 10 and 13, refer to Table 2) from the searched literature referred to community-based testing as a key strategy in improving HTS coverage among men, this being addressed through three categories: hotspot, door-to-door testing and mobile clinics.

Community-based testing, also known as community-based HTS, involves HCWs offering HIV testing outside of traditional health facilities at convenient times and place, such as in community settings, for example, ‘moonlight’, evening or weekend testing.^4^ This approach has shown to be effective in reaching individuals who have never been tested before and in identifying new HIV infections, particularly among men who may not typically seek testing. Evidence from four studies in this review indicated that more than half of the individuals tested through community-based HTS were men, most accepting the testing services when offered and being actively linked to treatment services thereafter.^4,7,18,20^

**Hotspots testing:** Targeted HIV testing by health facilities, NGOs or HCW can be effective in hotspots, these being places where people who may be at increased risk gather for specific activities, such as at bars, brothels, football grounds, video centres and truck stops.^18^ In the case of men who rarely attend healthcare facilities, they should be encouraged to test, with testing opportunities being made available where they gather being an effective approach to increasing uptake of HTS.^18^ To increase the uptake of testing, men’s leisure places and recreation ‘hot spots; can be targeted to provide holistic HTS.^7^

**Door-to-door testing (Home-based testing):** Sustained implementation of home-based testing and support for rapid treatment initiation has been shown to increase testing and treatment coverage among men in high-burden settings or hotspots.^4^ However, for such models to be effective, sustainability and resource needs must be considered, specifically, how men then access care services, including treatment.^4^ Index Testing Services (ITS), also known as partner notification or contact tracing, is an effective strategy for reaching men for case finding.^4^ Although there are challenges with following-up contacts for testing, this may be averted by implementing active testing at the partners’ homes, which is much more effective than the passive approach of waiting for them to present for testing.^7^ Healthcare workers can be trained by healthcare facility managers to offer ITS using motivational interviewing (MI) skills and to obtain the partner’s contact details to enable them to be actively tracked and offered HTS during door-to-door testing. A qualitative study conducted among 26 males in KwaZulu-Natal province, South Africa, found that most participants suggested that tailored and confidential HIV testing and treatment services could alleviate men’s fear and encourage them to utilise HIV care services. Home-based HIV testing through door-to-door initiatives was regarded as an effective approach to providing opportunities to test, deliver immediate results with counselling and actively link those who tested positive to the nearest clinic for treatment.^20^

**Mobile clinics:** Confidential community-based HIV testing and treatment services could include mobile clinics at places that were accessible and convenient for the people who wanted to use them.^20^ A descriptive qualitative study in the phenomenological tradition in seven public health facilities in Blantyre, Malawi, among men and HCWs suggested the use of mobile clinics in community settings. This included schools or places designated by the chief for service provision to increase the uptake of HTS and offer an opportunity to link those who test HIV positive to treatment services.^7^

#### Sub-theme 3: Workplace testing

Three studies (4, 10 and 13, refer to Table 2) from the researched literature mentioned workplace testing as a key strategy in improving HTS coverage among men, this being addressed through two categories: industrial or workplace-based testing and maintaining the confidentiality and avoiding stigma.

**Industrial or workplace-based testing:** Men prefer workplace-based HTS, which may be offered at their onsite clinics, because of its convenience and the absence of stigma associated with testing at a clinic. This option has been shown to be feasible and acceptable among men and assists with reaching those who may be reluctant to go to their local health facility.^7^ A qualitative study among affluent men in Tanzania revealed that in environments such as the workplace, they were able to publicly demonstrate their roles as ‘leaders’ by testing for HIV and encouraging others to do the same.^21^ Reaching men in their workplaces has the potential to change the current gendered health delivery system norms that have been negatively impacting HIV testing rates among men. It is also important to enhance HIV workplace policies to improve testing.^7^ Areas to be targeted to reach more men include farms, industries, truck stops and mines, with HIVST being an option in high HIV burden settings where most of the staff are men.^4^

**Maintain confidentiality and avoid stigma:** An independent cross-sectional survey in 2016–2017 and 2018 of men and HCWs (20–40 years old) in Eswatini (20–34 years old) and Durban, South Africa, found a need to maintain confidentiality and avoid any form of unintended disclosure, with stigma being an underlying factor that prevented people from attending clinics for testing.^22^ The HCWs corroborated the men’s views and discouraged providing services at locations associated with village chief’s to limit public exposure and the associated stigma and ensure confidentiality by providing them in neutral places with only male service providers.^22^

### Theme 2: Motivation and support

This theme consisted of two sub-themes: stakeholder involvement and creating a demand for testing. Tools and interventions that increase the demand for HTS are needed to reach people who are unaware of their options or unwilling to seek their use. In addition, peer-led interventions and the use of digital platforms and tools, such as social media and video-based messages, are promising and can be considered where feasible.^4^

#### Sub-theme 4: Stakeholders involvement

Two studies (5 and 6, refer to Table 2) from the researched literature referred to stakeholder involvement as a key strategy in improving HTS coverage among men, the two categories being faith-based organisations (FBO) and men’s health associations.

**Faith-based organisations:** Collaborating with FBOs to bridge the gap between ideological approaches to HIV and HTS proved successful in reaching men in South Africa, including older men, a hard-to-reach group. This was carried out through campaigns organised by the district health departments in collaboration with church leaders to reach a large proportion of first-time testers and improve access to services.^5^ Religious institutions (RIs) and FBOs continue to play an important role in responding to the HIV epidemic in Africa, as they have the potential to reach many people and to have a significant impact on the levels of HIV testing uptake, including for men, in what are traditionally conservative population.^5^

**Men’s health associations:** Men’s forums and societies, such as the South Africa Men’s Forum, where men meet monthly to discuss their issues and the Justice Men’s Forum, play an important role in HIV services. Participants described these gatherings as opportunities for them to discuss the challenges they face, learn positive coping strategies and promote HIV risk reduction and linkage to services.^22^ Collaboration with organisations that promote the uptake of men’s health services for a variety of conditions is essential for reaching more males for HTS.

#### Sub-theme 5: Create a demand for testing

Three studies (8, 9 and 13, refer Table 2) from the researched literature included creating a demand for testing as a key strategy in improving HTS coverage among men, the two categories that emerged being motivate and encourage men to conduct testing and share information about the importance of HTS.

**Motivate and encourage men to conduct testing:** To increase testing uptake among young men, providing incentives, such as free earphones, data bundles and USB flash drives, has been shown to help retention in HIV treatment and care. This enables healthcare providers, CHWs and community mobilisers to educate them about HIV/AIDS and HTS.^6^ Peer-led interventions and the use of digital platforms and tools, such as social media and video-based messages, show promise for increasing the uptake of testing and should be considered, where feasible, as mechanisms to encourage men to conduct testing for HIV. Digital tools can be especially appealing to younger men, including those from at-risk populations.^4^

Motivating men to seek HIV testing services and to remain on ART through an existing programme in South Africa, such as the Coach Mpilo and MINA health strategy, can assist in improving the 95-95-95 strategies.^23,24^ Coach Mpilo is a programme that utilises men living with HIV, known as ‘coaches’, to support and guide newly diagnosed men, as well as those who have returned to care after a treatment interruption, known as ‘players’. In addition, the coaches provide ongoing guidance and support, based on their personal experience of living with HIV, from the point of diagnosis or return to care to achieving treatment stability and viral suppression.^24^ The coaches help the men to view treatment as a means to gain control over HIV and to feel strong, healthy, safe, desirable and ultimately, normal again.^23^ They intend to help men live openly and confidently with HIV, free from the fear and shame that they may have initially felt.^24^

**Share information about the importance of HIV testing services:** Targeting outreach, education and community initiatives to raise awareness about HIV/AIDS and the importance of HTS can help to dispel misconceptions about being infected, while encouraging proactive health care-seeking behaviours that can be used to encourage testing.^6^ To encourage young men to conduct HTS, mobile health (mHealth) applications have been shown to address many of the barriers to HTS by encouraging the uptake of testing as well as accessing prevention packages, such as condoms, PrEP, counselling and treatment services through information sharing. These applications require promoting linkage to HIV-related counselling for ABYM, this being conducted in a respectful way that addresses the shame associated with being HIV positive, concerns about the future, issues of disclosure and leveraging social support.^11^

### Theme 3: Health facility services

This theme consisted of two sub-themes: facility-based testing and services provided by male HCWs. Health facilities continue to be important locations where men access HTS, and to increase testing uptake, it is important to routinely offer HTS to men when they attend health services for any reason, with facility-based HTS needing to be adapted to better reach men.^4^

#### Sub-theme 6: Facility-based testing

Four studies (4, 8, 12 and 13, refer Table 2) from the researched literature mentioned facility-based testing as a key strategy to improving HTS coverage among men, the two emerging categories being provider-initiated testing and counselling, and the provision of comprehensive HTS during facility visits.

**Offer provider-initiated testing and counselling:** Facility-based testing refers to HTS provided in a health facility or laboratory, at stand-alone HTS sites, also known as voluntary counselling and testing (VCT), or offered at every opportunity at clinical sites to all patients who present for any condition, this approach being known as provider-initiated testing and counselling (PITC).^4^ This means that rather than waiting for people to request an HIV test, clinicians offer it as part of the standard service that patients receive, irrespective of the reason for their visit during clinical consultation. Affluent individuals can access HTS through private clinics or healthcare appointments, which are paid for by health insurance schemes, or by traveling to other cities or countries to evade the stigma associated with local testing.^21^ Optimising facility-based HTS to make it accessible, inclusive and friendly to men can also contribute to improving their uptake of services.^4^ This includes reducing structural barriers and missed opportunities to offer testing, such as extending service hours, using rapid diagnostic tests to provide immediate results, and ensuring client confidentiality and stigma-free services.^4^

**Provide comprehensive HIV testing services during facility visit:** Health facilities remain essential for men to access HTS, particularly in areas with a high burden of HIV, and should be routinely offered to men when they visit facilities.^4^A descriptive, exploratory, qualitative study conducted among 17 young men and two healthcare providers in Ladysmith, KwaZulu-Natal province, identified unpleasant attitudes and inappropriate behaviour of healthcare providers offering HTS as a factor that discouraged men from accessing comprehensive HTS.^6^ Patient satisfaction surveys should be conducted routinely to identify barriers leading to inadequate access to healthcare facilities by men to enable remedial actions can be implemented timeously. Counsellors should offer HTS to men covering all streams including acute Tuberculosis (TB) and chronic follow-up. Men in high-burden settings can be motivated to attend facilities during their partner’s pregnancy.^4^ This strategy led to a 3-fold increase in the number of men who tested for HIV and helped to ensure that all the partners of the pregnant women who tested HIV positive were also tested and linked to appropriate services.^16^

#### Sub-theme 7: Services provided by male healthcare workers

Four studies (1, 2, 3 and 8, refer Table 2) from the researched literature referred to the provision of services by male healthcare providers as a key strategy to improving HTS coverage among men, this being addressed by two categories: providing male-friendly health services and providing services by male nurses and counsellors.

**Provide male friendly health services:** Male-only clinics or services were accessed by younger and healthier men compared to those who traditionally make use of health services, including for HTS.^25^ Men are reported to delay seeking health services as they perceive such spaces being for women, which means that they often leave their health problems until late in the condition.^6^ Having male-only clinics or services may ensure early access to testing and treatment for many conditions, including HIV, and prevent the avoidable morbidity and premature mortality that can result from late presentation. Such services also resulted in lower ART attrition rates than those that occur in the general population, as reported in a study in Khayelitsha, South Africa.^6^ These findings are similar to a study that supported clinic-specific adaptations to create more male-friendly environments.^25^

**Provide services by male nurses and counsellors:** There is a need to re-orient and sensitise clinical management to include more male nurses, clinicians and counsellors in clinical settings to provide services to men.^20^ A randomised controlled trial (RCT) conducted in Tanzania showed that training men as peer educators to engage other men in their networks in conversations about reducing inequitable gender norms was effective.^18^ This is particularly important in societies where it is not considered appropriate for men to discuss their health problems with women, which can result in delays in health-seeking behaviour.

## Implications and recommendations

This narrative review aimed to describe the interventions used to reach men through HTS opportunities at both facility and community levels to improve their access to treatment services. A literature review design and relevant methods were applied to identify strategies that can improve HTS uptake among men in both primary health care and community-based settings. This literature review found 13 articles to be relevant, and revealed that improved access to testing, motivation and support, and health facility services are key interventions used to reach men to improve HTS uptake. These findings are similar with the study conducted by Dovel which reported that convenient services, actively engaging men and providing positive experiences with health services, improves health utilisation.^26^ This review focused on interventions that can be implemented in SSA to improve HTS among men where there is a high rate of HIV, noting that those interventions can also be applicable in other parts of the world.

The findings revealed that HIVST, community-based testing and workplace testing are key interventions for reaching men through HTS, with HIVST being regarded as a safe, accurate and acceptable approach.^4^ The HIV self-testing can be utilised to reach men who are unable or unwilling to access healthcare facilities for testing in hard-to-reach areas with limited resources and where mobile clinics are unable to frequently reach the community for support. Additional research in developing countries is needed, including on its potential population-level impact, cost-effectiveness, methods of delivery and linkage to HIV prevention and care; with the model remaining a promising alternative to healthcare provider-delivered HIV testing.^27^ Ensuring that there is a stringent mechanism to follow up HIVST results remains a critical area to be investigated through research and supported by HCWs through implementation.^27^

Men in SSA have also been reached for HIV testing using HIV and AIDS sensitisation campaigns, clinician referrals, home- and community-based HIV testing, incentives for HIV testing, partner (index) testing and traditional HIV testing methods.^7^ Strengthening referral pathways between community organisations and healthcare facilities to ensure access to care for men is critical.^27^ This was noticed by Ndlovu, who found that targeted outreach, education and community initiatives were necessary and effective for raising awareness about HIV and AIDS, highlighting the importance of HTS, as well as dispelling misconceptions and encouraging proactive healthcare-seeking behaviour among men.^6^

In an effort to reach more men for testing some interventions studies found that the proportion of individuals at home who were male was low resulting in the limited impact of population level testing to provide services for men.^27^ This is particularly relevant in areas of high male mobility because of employment or not being at home during the day for a variety of reasons.^27^ Strengthening community-based testing services, including home testing, by allocating and monitoring targets for HCWs, could improve men’s access to HTS. Hensen found that community-based programmes improved HTS among men, with a need to plan resource allocation to increase its uptake.^27^ Combined with behavioural interventions, this approach is more effective for increasing the uptake of HTS and other services than the conventional health facilities-based testing and counselling approaches on their own.^28^ These findings reinforced previous findings indicating that community testing was an evidence-based strategy that can be implemented, in addition to raising serostatus awareness through health education, also this strategy has the potential to lower high-risk behaviour and HIV transmission.^26,27,29^

Mobile HTS is a popular community-based strategy that has become well-liked because of its practicality and accessibility in reaching more men for HTS, especially in hard-to-reach areas. In contrast to facility-based HTS, mobile services enable people to be tested at places and times that suit them, and meet their needs for dignity, privacy and shorter waiting times.^6^ According to the WHO, reducing structural barriers to accessing treatment and missed opportunities to offer testing, using rapid diagnostic tests to provide immediate results, improving client confidentiality and assuring stigma-free services for key populations, including men, are essential service elements.^4^

Testing can be conducted in high HIV-burden workplaces where men may be at risk, such as mines, truck stops, farms, industrial sites and taxi ranks.^4^ Targeting workers who are not able to reach health facilities because of work commitment is key to improving HTS uptake. However, even if HTS is fully integrated with employment settings, large numbers of men will not have access to workplace HTS in settings where formal employment is low.^27^ In a qualitative study conducted in Cape Town, HCWs felt that there were significant gaps in men’s engagement with HIV care, and identified masculine gender norms, the persistent impact of HIV stigma and competing priorities of employment as key barriers.^30^ Furthermore, an integrated approach that can be implemented combining testing and treatment with other HIV interventions such as Voluntary Male Medical Circumcision (VMMC), PrEP and chronic disease screening can increase intervention programme efficiency while reducing HIV-related stigma among men.^12^

In order to delimit stigma around HIV, there is a need to provide HCWs sensitisation training and job aids on how to interact with men as clients either at healthcare facilities or during workplace testing. It is well known that misconception and implicit bias arise from lack of understanding, experience and professional skills or tools to engage men.^26^ Men’s HTS uptake is often associated with women’s willingness to undertake the tests and adherence to Vertical Transmission and Prevention of Communicable Infection (VTPCI), men’s HIV-testing is crucial to the success of VTPCI interventions and for women’s health in general.^27^

Studies have shown that stakeholder involvement and creating a demand for testing can play an important role in promoting HTS among men, which can be done through regular contact with men who are members of formal societies. The NDoH in, collaboration with NGOs, should involve key stakeholders, such as RL, FBOs and men’s associations, to reach men for testing by conducting regular community dialogues and addressing health issues during church services and events. Engaging religious institutions to encourage HIV testing by the men in their congregations and communities is another understudied strategy to increase men’s access to HTS.^5^ In response to the HIV epidemic in Southern Africa, churches have typically focused on prevention messaging, as well as counselling and providing support and care for individuals who are infected, affected or impacted by the disease.^5^

Creating a demand for testing is also important and highlights the need for HCWs to motivate and encourage men to conduct testing through information sharing about the importance of HTS, this being significant for improving uptake. Behavioural interventions, such as health promotion activities, are effective in increasing the uptake of HTS among men, including through health education and distributing information, education and communication (IEC) material in places where men can be reached.^28^ This is similar to the findings from the study performed by Sharma, that targeted messaging to motivate men (e.g., protection of sexual partners and future children, restoration of health through ART) can increase testing uptake and linkage.^12^ Men consistently described welcoming the opportunity to participate in HIV prevention programming, which ranged from a few brief sessions to multiple intensive meetings held over many weeks.^22^

Measures such as facility-based testing and service provision by male HCWs have been shown to improve HTS uptake among men.^4^ Offering PITC and providing comprehensive HTS during facility visits plays an important role in ensuring that they can access these services.^31^ Provider-initiated testing and counselling at all service points in health facilities is essential for increasing HTS among men, and the WHO recommends that it be offered at clinical sites in all streams within health facilities as a way of encouraging testing among those who may not actively seek out such tests.^4^

Facility-based testing has shown great strides in reaching men who access health services, and although PITC is unlikely to increase men’s population levels of HIV testing, the strategy can reach a high proportion of men already seeking services.^27^ Inadequate access to youth-friendly health services prevents ABYM from seeking HIV testing, treatment and prevention services.^32^ Establishing and expanding youth-friendly spaces and services with confidential and non-judgemental staff is essential.

Improving social support and providing poverty alleviation can change norms around HTS and access to treatment while addressing other factors contributing to low engagement in care among men, for example food insecurity and poverty.^12^ Addressing the root cause for low HTS uptake needs a team’s approach including different sectors and departments. A multipronged approach to HTS may ensure a more comprehensive defence against HIV transmission to other populations while interrupting gendered norms that are harmful to the well-being of ABYM, men and people of all genders. Furthermore, by implementing these measures, we can work towards creating an HIV-free future for the youth of SSA.^32^

The literature review identified male-friendly health services and services provided by male nurses and counsellors, as being important to assist men in accessing HTS. While this intervention may improve HTS uptake among men, special consideration should be given to engaging male HCWs in the delivery of health and social services to ABYM. This may require careful attention regarding how to support ABYM to feel safe, while not upholding rigid gender binaries and harmful masculine norms because of the separation of services.^32^

No single strategy will probably be adequate to enhance men’s engagement in HIV services, indicating a need for more comprehensive thought regarding a set of strategies that are both feasible and sustainable.^27^ These strategies should aim to improve men’s participation in care, not only in the context of HIV but across all areas of healthcare where male engagement is lacking. While there is a necessity for research, policy initiatives and interventions tailored to male-specific services, we must also focus on transforming the existing healthcare services, which are predominantly staffed by women, to better and sustainably increase the supply, access and quality of healthcare services for men.^27^

Evidence that has been compiled arising from this review may motivate and support decision-making by the HTS implementers in countries where men’s testing level is low in developing strategies that can be implemented in healthcare facilities to target men for comprehensive service provision.

### Limitations

A number of limitations may have affected the study, including the small sample size, with less stringent inclusion criteria possibly enabling more studies to be reviewed. While the study focused on existing approaches that can be implemented to improve testing uptake among men, other key indicators, such as viral load uptake and missed appointments in the HIV programme, were not covered in the study. The review did not include sources such as newspaper articles, theses and books, which might provide useful insight into the topic under investigation. Lastly, the inclusion of studies in languages other than English may have resulted in other approaches being identified.

### Recommendations

The literature review indicates the need for more studies into coordinated, multifaceted approaches to promote the uptake of HTS among men. Improving their access to accurate, consistent and easily accessible HTS information as a means to encouraging testing can be performed by utilising local radio stations, community newspapers, billboards and social media platforms. Home-based testing, through HIVST or door-to-door efforts, can reach men who are unable or unwilling to test, and for those who are unable to test and receive their results immediately, the results can be followed up within 72 h by HCWs over the phone. Social Network Testing, which involves testing people who are closely related and belong to the same social group, can improve reaching the hard-to-reach male population. Healthcare services for men need to be available from HCWs they can relate to, specifically men. Strategies that target men, such as Coach Impilo and Mina Health for Men, could be implemented in healthcare facilities with the assistance of civil societies, men’s forums and other stakeholders.^8,23^ Index or partner testing needs to be included in all circumstances where a partner is indicated to reach males for testing.^4^

## Conclusion

Approaches for reaching men for HTS need to include multifaceted strategies that combine facility and community-based testing methods to encourage those who are positive to access treatment at health facilities or registered clinics.^4,20^ Those who test negative but have the potential to contract the virus also need to test regularly, the intention being to ensure that all those who are infected can access treatment, reduce their viral loads and limit its onward transmission. There is a need to implement innovative approaches to normalise testing for men as part of routine healthcare provision and for ART uptake and adherence to be regarded the same as medication for other chronic conditions.^3^ Through the coordinated implementation of several strategies, HTS uptake gaps among males can be improved, leading to reduced morbidity and premature mortality that is associated with late presentation and failure to initiate ART.^4^Providing positive experiences with health services largely improves service utilisation among men leading to increase in testing uptake in healthcare facilities.^25^
